# Can an Internet-Based Health Risk Assessment Highlight Problems of Heart Disease Risk Factor Awareness? A Cross-Sectional Analysis

**DOI:** 10.2196/jmir.2369

**Published:** 2014-04-18

**Authors:** Justin B Dickerson, Catherine J McNeal, Ginger Tsai, Cathleen M Rivera, Matthew Lee Smith, Robert L Ohsfeldt, Marcia G Ory

**Affiliations:** ^1^School of Public HealthDepartment of Health Promotion and Community Health SciencesTexas A&M Health Science CenterCollege Station, TXUnited States; ^2^Department of Internal MedicineScott & White HealthcareTemple, TXUnited States; ^3^College of MedicineTexas A&M Health Science CenterCollege Station, TXUnited States; ^4^College of Public HealthDepartment of Health Promotion and BehaviorThe University of GeorgiaAthens, GAUnited States; ^5^School of Public HealthDepartment of Health Policy & ManagementTexas A&M Health Science CenterCollege Station, TXUnited States

**Keywords:** health risk assessment, Internet, risk factors, health disease, concordance

## Abstract

**Background:**

Health risk assessments are becoming more popular as a tool to conveniently and effectively reach community-dwelling adults who may be at risk for serious chronic conditions such as coronary heart disease (CHD). The use of such instruments to improve adults’ risk factor awareness and concordance with clinically measured risk factor values could be an opportunity to advance public health knowledge and build effective interventions.

**Objective:**

The objective of this study was to determine if an Internet-based health risk assessment can highlight important aspects of agreement between respondents’ self-reported and clinically measured CHD risk factors for community-dwelling adults who may be at risk for CHD.

**Methods:**

Data from an Internet-based cardiovascular health risk assessment (Heart Aware) administered to community-dwelling adults at 127 clinical sites were analyzed. Respondents were recruited through individual hospital marketing campaigns, such as media advertising and print media, found throughout inpatient and outpatient facilities. CHD risk factors from the Framingham Heart Study were examined. Weighted kappa statistics were calculated to measure interrater agreement between respondents’ self-reported and clinically measured CHD risk factors. Weighted kappa statistics were then calculated for each sample by strata of overall 10-year CHD risk. Three samples were drawn based on strategies for treating missing data: a listwise deleted sample, a pairwise deleted sample, and a multiple imputation (MI) sample.

**Results:**

The MI sample (n=16,879) was most appropriate for addressing missing data. No CHD risk factor had better than marginal interrater agreement (κ>.60). High-density lipoprotein cholesterol (HDL-C) exhibited suboptimal interrater agreement that deteriorated (eg, κ<.30) as overall CHD risk increased. Conversely, low-density lipoprotein cholesterol (LDL-C) interrater agreement improved (eg, up to κ=.25) as overall CHD risk increased. Overall CHD risk of the sample was lower than comparative population-based CHD risk (ie, no more than 15% risk of CHD for the sample vs up to a 30% chance of CHD for the population).

**Conclusions:**

Interventions are needed to improve knowledge of CHD risk factors. Specific interventions should address perceptions of HDL-C and LCL-C. Internet-based health risk assessments such as Heart Aware may contribute to public health surveillance, but they must address selection bias of Internet-based recruitment methods.

## Introduction

The Framingham Heart Study defines cardiovascular disease (CVD) as a combination of coronary heart disease (CHD), various types of stroke, peripheral artery disease, and heart failure [1]. CVD is a leading cause of death in both males and females living in the United States [2]. In 2006, CVD was responsible for the death of 1 in 4 Americans [3]. CVD is one of the costliest medical conditions to treat with total economic costs estimated at more than US $300 billion annually [3], a cost that is also predicted to rise substantially over the next 2 decades as a result of technological improvements in care coupled with minimal reduction in the prevalence of CVD [4].

Lifestyle risk factors, including tobacco and alcohol use, poor diet, lack of exercise, and obesity [5], as well as a genetic predisposition to problems such as familial hypercholesterolemia [6] contribute to the high prevalence of CVD. To facilitate prevention of CVD, it is important to measure CVD risk factors on a regular basis. In an effort to reduce CVD mortality, the National Heart Lung and Blood Institute (NHLBI) launched The Heart Truth campaign to raise awareness of CVD risk factors [7]. A year later, the American Heart Association (AHA) adopted the Red Dress symbol and launched its own campaign, Go Red for Women to emphasize the importance of knowing and reducing CVD risk factor values among at-risk females in accordance with clinical guidelines [8].

Patient engagement in appropriate screening and risk factor modification by health care providers is critical in preventing CVD. For example, it has been shown that a key element of dyslipidemia, a low value of high-density lipoprotein cholesterol (HDL-C), is largely an unknown CVD risk factor among the general public [9]. Similar awareness of elements of dyslipidemia has also been shown to vary across different populations [10]. As a result, it is important for patients being screened to be provided with education on CVD risk factors and CVD risk factor modification.

Self-report of risk factors often guides epidemiological studies of disease prevalence [11]. As a result, it is vitally important to establish the accuracy of self-reported values against clinically measured values. Several recent studies have called the accuracy of self-reported data into question, especially for CVD risk factors [11-13]. Some studies have also produced discordant results about the accuracy of self-reported risk factor data for different socioeconomic groups and different geographic locations [14,15]. There has been a general lack of public health investigation of the agreement of self-reported and clinically measured CVD risk factors.

This study has three aims. First, it examines the degree of agreement between self-reported and clinically measured risk factors for CHD (the most prevalent CVD condition representing half of all cardiovascular diseases), including total cholesterol (TC), HDL-C, low-density lipoprotein cholesterol (LDL-C), systolic blood pressure (SBP), diastolic blood pressure (DBP), body mass index (BMI), and diabetes mellitus (DM) status, to understand which risk factors are most accurately reported by community-dwelling adults [16]. Second, it analyzes agreement of self-reported and clinically measured CHD risk factor values according to the Framingham Heart Study’s 10-year CHD risk model [17] to determine if those at higher risk of CHD have a greater understanding of their CHD risk factors versus those at lower risk of CHD (ie, the resulting kappa statistics of self-reported and clinically measured agreement are stratified by Framingham 10-year CHD risk). Finally, because self-reported data often present missing data challenges, the study examines whether the method of accounting for missing data influences the results.

## Methods

### Heart Aware Cardiovascular Health Risk Assessment

Heart Aware is a cardiovascular health risk assessment tool offered by Navigant Consulting, Inc (Navigant Consulting, Inc, Chicago, IL, USA). The data used in this study came from Heart Aware assessments conducted at 127 clinical sites across the United States between January 1, 2006 and June 30, 2010. Research approval for the data was granted by the Institutional Review Board of Texas A&M University. The risk assessment was administered through the Internet. The assessment began by asking respondents, who voluntarily accessed the survey, a series of self-identifying demographic questions about their race, sex, and age. These respondents were recruited to the survey through marketing activities of the hospitals sponsoring the Heart Aware assessment, such as television media advertising campaigns and print media placed throughout both inpatient and outpatient facilities. Respondents were then asked to report their height, weight, whether they used tobacco, level of physical activity, DBP, SBP, TC, HDL-C, and LDL-C. Respondents were then asked to report the last time their health care provider measured their blood pressure, cholesterol, and checked their diabetes status. Finally, respondents were asked a series of questions about their family history, current medications, and current health history, with specific emphasis on CHD symptoms or diagnoses. As the respondents moved through the assessment, 2 unique tools reported data back to the respondents. First, as questions were answered by the respondents, a visual scale indicating the risk for CHD attributable to each risk factor was displayed. Scales for each risk factor were indicated on a color spectrum from green (low risk) to red (critical risk), and were updated as additional information was provided by the respondent. Second, as the respondents moved through different categories of questions, the tool provided the respondents with education about CHD and associated risk factors. This occurred in the form of text boxes on response pages. Definitions of medical terms were also provided to enhance the respondents’ knowledge of CHD and related risk factors.

The clinical sites that elected to offer the Heart Aware assessment determined the level of risk that would prompt the clinical site to extend an invitation to the respondent for a free on-site clinical risk assessment in which their self-reported CHD risk factor values would be measured by a clinician for comparison and validation. Each site set its own criteria for inviting participants to the free clinical assessment; Navigant did not document this protocol, including the method of extending the invitation to the participants. Anecdotal evidence suggests respondents were more likely to be invited for a clinical assessment if their self-reported values indicated 2 or more CHD risk factors.

### Coronary Heart Disease Risk Factors

The variables analyzed as CHD risk factors were collected from the Heart Aware risk factor assessment. These included self-reported and clinically measured values of TC, HDL-C, LDL-C, SBP, DBP, BMI, as well as DM status and tobacco use. These variables were reported in the clinically assessed dataset as ordinal scales based on their frequencies and clinical guideline ranges. This resulted in ordered categories (referred to as “ranges” below) within each variable as follows: TC (<160 mg/dL, 160-199 mg/dL, 200-239 mg/dL, 240-279 mg/dL, >279 mg/dL), HDL-C (<35 mg/dL, 35-44 mg/dL, 45-49 mg/dL, 50-59 mg/dL, and >59 mg/dL), LDL-C (<100 mg/dL, 100-129 mg/dL, 130-159 mg/dL, 160-189 mg/dL, and >189 mg/dL), SBP (<120 mm Hg, 120-129 mm Hg, 130-139 mm Hg, 140-159 mm Hg, 160-199 mm Hg, and >199 mm Hg), and DBP (<80 mm Hg, 80-84 mm Hg, 85-89 mm Hg, 90-99 mm Hg, 100-114 mm Hg, and >114 mm Hg). DM status and tobacco use were coded as yes or no responses.

### Sample Selection Criteria

Respondents who provided a self-reported health assessment and were chosen for and participated in a free clinical assessment of their CHD risk factors were eligible for inclusion in the sample. Those with a prior history of CVD- and CHD-related procedures, such as stroke, acute myocardial infarction, abdominal aortic aneurysm, cardiac arrest, congestive heart failure, angioplasty, catheterization and stent procedures, heart bypass, and carotid procedures, were excluded from the sample to maintain the integrity of the study objective which was to evaluate how community-dwelling adults who had not received a diagnosis of CHD viewed their risk relative to their actual clinically measured risk for CHD.

Depending on the preferred strategy for addressing missing data, 3 samples were available for analysis. To maximize our understanding of the research questions in the study, all 3 samples were made available for analysis. First, analyses were conducted on the original dataset. Given default settings in Stata version 12 (Stata Corp LLP, College Station, TX, USA), this resulted in a listwise deleted sample. Second, analyses were conducted on the original dataset, but with a change in the default settings. Instead of eliminating cases missing any of the variables being analyzed (as was the case with the listwise deleted sample), cases were only removed on a variable-by-variable analysis basis. This resulted in a pairwise deleted sample. Finally, analyses were conducted on the imputed sample. Results of all analyses performed on all 3 samples were then examined.

### Self-Assessment Missing Data

Self-reported health status data are known to contain substantial amounts of missing data [18]. Participants often choose not to answer certain questions for a variety of reasons, such as lack of knowledge, time constraints in answering the survey, or a desire not to answer certain questions based on individual preferences. Missing data introduces many analytical challenges, especially relating to biased statistical estimators when making inferences from data [19,20]. To address missing data issues, a 3-step process was used to evaluate the missing data. First, data were analyzed for the degree of missing data, such as the number of missing responses across individual variables and individual cases. Second, the pattern of missing data was analyzed using the “mvpatterns” user-written command [21] in Stata version 12. This command allowed the researcher to determine whether the pattern of missing data was missing completely at random (MCAR), missing at random (MAR), or missing not at random (MNAR) [20]. Third, based on the result of the first 2 steps noted previously, a methodology was devised to adjust the dataset where appropriate to account for missing data where it was deemed a concern. Several techniques were evaluated for this purpose such as listwise deletion and multiple imputation. Multiple imputation was selected as the preferred method for addressing self-reported missing data because of the large number of missing responses and MAR-identified pattern of missing data discussed later in this paper. Multivariate normal imputation with 5 imputations was used to impute missing values of SBP, DBP, and TC because these variables were most closely aligned with the Framingham 10-year CHD risk model. TC was analyzed because it had more complete self-reported data than HDL-C and LDL-C. These latter variables were key considerations when analyzing missing values in the clinically measured dataset discussed subsequently. Multivariate imputation was chosen because of its ability to take advantage of all variables in the analysis to impute the selected missing variables [22]. Five imputations were selected to balance statistical rigor with processing speed. Independent variables used to impute were selected based on the completeness of data and their theoretical association with the imputed variables. The independent variables used for imputation were age, rural/urban designation, sex, DM status, and BMI range.

The analysis of missing data for the self-reported data was vital to the integrity of the study. Because the self-reported values were used as the basis for choosing individuals for a free clinical assessment, missing data could have profound implications for how participants were selected, possibly resulting in selection bias. Because the clinical sites set their own selection criteria that were not chronicled by Navigant, it was even more important to examine whether the missing self-reported data influenced selection by the clinical sites. This test for selection bias in the clinical assessment process was conducted using the original dataset and the imputed dataset. Using both datasets individually, the analysis was done by examining differences in risk factor variable means between those individuals selected for clinical assessment and those individuals not chosen for clinical assessment. For this purpose, *t* tests with statistical significance determined at the alpha=.05 level were used.

### Clinical Assessment Missing Data

Although the statistical challenges of missing data noted for the self-reported data also applied to the clinically measured data, there were additional complexities that necessitated a separate analysis of the clinically measured dataset. First, when analyzing clinically measured data, many of the variables used in the Framingham 10-year CHD risk model were reported as ranges by the clinical sites. These ranges corresponded to the ranges used in the Framingham 10-year CHD risk model. Second, the pattern of missing data in the clinically measured dataset was different than the self-reported dataset. As such, methodologies used to address missing data concerns in the clinically measured dataset had to recognize both unique patterns of missing data and the fact the data were now reflected in ranges unlike individual values found in the self-reported dataset. Given the MAR-identified pattern of missing data discussed later in this paper, missing data in the clinically measured dataset was imputed with an ordered logistic model versus the multivariate normal model discussed previously. The ordered logistic imputation was carried out with 5 imputations and was used to impute missing values for ranges of SBP, DBP, TC, HDL-C, and LDL-C because these variables were most closely aligned with the Framingham 10-year CHD risk model. Note the imputation of the clinically measured dataset imputed HDL-C and LDL-C in addition to TC. This was because these variables provided a more detailed view of dyslipidemia relative to TC. It was possible to do these imputations in the clinically measured dataset because there was not as much missing data relative to the self-reported dataset. Independent variables used to impute were selected based on the completeness of data and their theoretical association with the imputed variables. The independent variables used for imputation were age, rural/urban designation, sex, DM status, and BMI range.

### Descriptive Statistics and Kappa Coefficients of Interrater Agreement

Descriptive statistics were conducted on each of the 3 samples. The following variables were dichotomized by sex: age, race/ethnicity, rural/urban designation, SBP, DBP, TC, HDL-C, LDL-C, DM status, and tobacco use. Continuous variables were measured with *t* statistics, and categorical variables were measured with chi-square statistics. Descriptive analyses were performed using Stata version 12. Test statistics were measured for statistical significance at the alpha=.05 level of statistical significance.

Weighted kappa coefficients of interrater agreement between self-reported and clinically measured CHD risk factors were calculated for males and females. A weighted kappa coefficient was used instead of an unweighted kappa coefficient to account for the degree of discordance between each self-reported and clinically measured observation based on the fact the data were categorized as ordinal ranges. The weighting procedure was performed in Stata version 12 using the “wgt(w)” extension of the “kapci” command. The weighted kappa procedure included 100 repetitions. Each coefficient was reported with its standard error and bias-corrected 95% confidence interval. According to established literature [23], the strength of interrater agreement of the kappa coefficient is described as poor (κ<.00), slight (κ=.00-.20), fair (κ=.21-.40), moderate (κ=.41-.60), substantial (κ=.61-.80), and almost perfect (κ=.81-1.00).

### Framingham Heart Study’s 10-Year CHD Risk Model

The 10-year CHD risk model from the Framingham Heart Study [17] was used to calculate each respondent’s 10-year CHD risk based on their clinically measured risk factor values. The weighted kappa coefficients were then calculated for each stratum of 10-year CHD risk scores to allow for an evaluation of risk factor agreement by level of CHD risk.

## Results

### Self-Reported Data, Missing Data, and Participant Selection

The Heart Aware cardiovascular health risk assessment was taken by 373,085 individuals. The clinical sites provided a clinical assessment to 22,346 (5.99%) of these individuals (note the number of individuals offered an assessment but not taking an assessment was not recorded). Among those responding to the self-reported assessment, 238,081 (63.81%) of the respondents did not answer at least 1 of the risk factor variable questions. Based on the robust information presented in the tables subsequently, extended analysis of the results is provided in the discussion section in this paper. Table 1 reports the results of the nonresponse rate for each risk factor variable in the self-reported dataset and in the imputed self-reported dataset.

**Table 1:** Missing data from the self-reported dataset (n=373,085) and the self-reported imputed dataset (n=373,085).

Variables by dataset		Complete, n	Missing, n	Total, n (n=373,085)	% Missing
**Self-reported dataset**					
	Sex	373,062	23	373,085	0.01%
	Age	348,065	25,020	373,085	6.71%
	Tobacco use	296,047	77,038	373,085	20.65%
	Diabetes status	370,388	2697	373,085	0.72%
	Systolic blood pressure	265,693	107,392	373,085	28.78%
	Diastolic blood pressure	260,714	112,371	373,085	30.12%
	Total cholesterol	189,554	183,531	373,085	49.19%
	Complete cases^a^	135,004	238,081	373,085	63.81%
**Self-reported imputed dataset**					
	Sex	373,062	23	373,085	0.01%
	Age	348,065	25,020	373,085	6.71%
	Tobacco use	296,047	77,038	373,085	20.65%
	Diabetes status	370,388	2697	373,085	0.72%
	Systolic blood pressure^b^	345,990	27,095	373,085	7.26%
	Diastolic blood pressure^b^	345,948	27,137	373,085	7.27%
	Total cholesterol^b^	345,569	27,516	373,085	7.38%
	Cases with all variables^b^	269,772	103,313	373,085	27.69%

^a^Not missing any variables.

^b^Variables were imputed.

Table 2 reports the results of the test of difference in means for the risk factor variables of those selected for clinical assessment from the self-reported dataset. It should be noted the table compares the clinically measured dataset for each variable unadjusted by imputation methods (ie, each self-reported variable has its own sample size). The sample size and very limited amount of missing data for each variable can be examined in Table 3. Table 3 also reports the same type of information as Table 2, but for those selected for clinical assessment adjusted by imputation methods. It is clear from both these tables that those with clinically measured risk factors had significantly higher values (ie, poorer values) than the overall self-reported population. This confirms the recruitment strategy of the clinics to find those at high risk of CHD and invite them for a free clinical screening of their risk factors.

**Table 2:** Self-reported and self-reported imputed versus clinically measured dataset: difference in means on critical risk factor values.

Variables by dataset		Sample size	Dataset, mean (SD)		*t* (*df*)^a^	*P*
			Self-reported	Clinically measured		
**Self-reported**						
	Systolic blood pressure	265,693	127.9 (12.1)	134.1 (13.2)	–72.3 (287,545)	<.001
	Diastolic blood pressure	260,714	82.3 (6.8)	85.1 (7.0)	–58.2 (282,495)	<.001
	Total cholesterol	189,554	193.0 (35.6)	205.6 (30.2)	–49.5 (210,644)	<.001
**Self-reported imputed**						
	Systolic blood pressure	345,990	127.8 (12.2)	132.4 (13.1)	–54.3 (368,243)	<.001
	Diastolic blood pressure	345,948	82.4 (6.8)	83.9 (7.0)	–31.8 (368,187)	<.001
	Total cholesterol	345,569	192.9 (35.5)	200.9 (33.4)	–32.6 (367,622)	<.001

^a^Pooled degrees of freedom.

### Missing Clinical Assessment Data

Clinically measured risk factor values were reported for 22,346 individuals. This dataset consisted of clinically measured data that were expressed as ordinal (ie, ranges of clinical values) and binary data (eg, DM status and tobacco use). Among these individuals, 6423 (28.74%) of the respondents did not answer at least 1 of the risk factor variable questions.

Table 3 reports the results of the nonresponse rate for each risk factor variable in the clinically measured dataset, including the self-reported nonresponse rate for the same respondents. Table 3 also reports the results of the nonresponse rate for each risk factor variable in the imputed clinically measured dataset.

**Table 3:** Missing data for the clinically measured dataset (n=22,346) and the clinically measured imputed dataset (n=22,346).

Variables in dataset		Clinically measured dataset			Clinical measured imputed dataset		
		Complete, n	Missing, n	% Missing	Complete, n	Missing, n	% Missing
Sex		22,364	0	0.00%	22,364	0	0.00%
**Age**		22,011	335	1.50%	22,011	335	1.50%
	Age (self-reported)	22,011	335	1.50%	22,011	335	1.50%
**Tobacco use**		22,346	0	0.00%	22,346	0	0.00%
	Tobacco use (self-reported)	22,346	0	0.00%	22,346	0	0.00%
**Diabetes status**		22,346	0	0.00%	22,346	0	0.00%
	Diabetes status (self-reported)	22,346	0	0.00%	22,346	0	0.00%
**Systolic blood pressure ranges** ^a^		21,854	492	2.20%	22,255	91	0.41%
	Systolic blood pressure ranges (self-reported)^a^	19,465	2881	12.88%	21,981	365	1.63%
**Diastolic blood pressure ranges** ^a^		21,783	563	2.52%	22,241	105	0.47%
	Diastolic blood pressure ranges (self-reported)^a^	19,268	3078	13.76%	21,951	395	1.77%
**HDL-C ranges** ^a^		20,105	2241	10.02%	21,850	496	2.22%
	HDL-C ranges (self-reported)^a^	9675	12,671	56.66%	20,595	1751	7.83%
**LDL-C ranges** ^a^		16,594	5752	25.72%	21,582	764	3.42%
	LDL-C ranges (self-reported)^a^	7829	14,517	64.91%	20,480	1866	8.34%
**Total cholesterol ranges** ^a^		21,092	1254	5.61%	22,055	291	1.30%
	Total cholesterol ranges (self-reported)^a^	14,144	8202	36.68%	21,287	1059	4.74%
**Cases with all variables** ^a^		15,923	6423	28.72%	21,241	1105	4.94%
	Cases with all variables (self-reported)^a^	7175	15,171	67.84%	20,263	2083	9.31%

^a^These variables in imputed dataset were imputed.

### Clinical Assessment Data, Descriptive Statistics: Listwise Deletion, Pairwise Deletion, and Imputed Samples

Table 4 reports the differences in means and proportions of the CHD risk factor variables by sex within the listwise deleted sample (n=5951).

Table 5 reports the differences in means and proportions of the risk factor variables by sex within the pairwise deleted sample (note the sample size varies by each risk factor variable as indicated in the table).

Table 6 reports the differences in means and proportions of the risk factor variables by sex within the imputed sample (n=16,879).

**Table 4:** Listwise deleted sample (n=5951): differences in means and proportions by sex.

Variables in dataset			Male (n=1938)	Female (n=4013)	Total (n=5951)	*t* (*df*)	χ^2^(*df*)	*P* ^a^
Age, mean (SD)			55.5 (10.3)	56.3 (11.2)	55.8 (10.6)	–2.6 (5949)		.008
**Race/ethnicity, n (%)**							32.4 (5)	<.001
	Non-Hispanic white		1678 (86.58)	3435 (85.60)	5113 (85.92)			
	African-American		114 (5.88)	335 (8.35)	449 (7.54)			
	Hispanic		42 (2.17)	112 (2.79)	154 (2.59)			
	Asian/Pacific Islander		68 (3.51)	67 (1.67)	135 (2.27)			
	American Indian/Alaskan Native		3 (0.15)	8 (0.20)	11 (0.18)			
	Other		33 (1.70)	56 (1.40)	89 (1.50)			
**Tobacco use, n (%)**							33.3 (2)	<.001
	Never		1248 (64.40)	2879 (71.74)	4127 (69.35)			
	Quit		525 (27.09)	870 (21.68)	1395 (23.44)			
	Yes		165 (8.51)	264 (6.58)	429 (7.21)			
**Diabetes, n (%)**							7.3 (4)	.12
	No		1726 (89.06)	3653 (91.03)	5379 (90.39)			
	Type 1		16 (0.83)	29 (0.72)	45 (0.76)			
	Type 2		184 (9.49)	316 (7.87)	500 (8.40)			
	Borderline		7 (0.36)	11 (0.27)	18 (0.30)			
	Unsure		5 (0.26)	4 (0.10)	9 (0.15)			
**Systolic blood pressure ranges (mm Hg), n (%)**							90.8 (5)	<.001
	<120		469 (24.20)	1386 (34.54)	1855 (31.17)			
	120-129		531 (27.40)	1127 (28.08)	1658 (27.86)			
	130-139		461 (23.79)	689 (17.17)	1150 (19.32)			
	140-159		394 (20.33)	666 (16.60)	1060 (17.81)			
	160-199		79 (4.08)	144 (3.59)	223 (3.75)			
	>199		4 (0.21)	1 (0.02)	5 (0.08)			
	**Self-reported**						147.1 (5)	<.001
		<120	360 (18.58)	1276 (31.80)	1636 (27.49)			
		120-129	750 (38.70)	1514 (37.73)	2264 (38.04)			
		130-139	546 (28.17)	750 (18.69)	1296 (21.78)			
		140-159	235 (12.13)	406 (10.12)	641 (10.77)			
		160-199	45 (2.32)	66 (1.64)	111 (1.87)			
		>199	2 (0.10)	1 (0.02)	3 (0.05)			
**Diastolic blood pressure ranges (mm Hg), n (%)**							107.7 (5)	<.001
	<80		829 (42.78)	2206 (54.97)	3035 (51.00)			
	80-84		550 (28.38)	1060 (26.41)	1610 (27.05)			
	85-89		253 (13.05)	356 (8.87)	609 (10.23)			
	90-99		254 (13.11)	323 (8.05)	577 (9.70)			
	100-114		47 (2.43)	67 (1.67)	114 (1.92)			
	>114		5 (0.26)	1 (0.02)	6 (0.10)			
	**Self-reported**						82.7 (5)	<.001
		<80	764 (39.42)	2029 (50.56)	2793 (46.93)			
		80-84	657 (33.90)	1251 (31.17)	1908 (32.06)			
		85-89	306 (15.79)	445 (11.09)	751 (12.62)			
		90-99	175 (9.03)	240 (5.98)	415 (6.97)			
		100-114	31 (1.60)	44 (1.10)	75 (1.26)			
		>114	5 (0.26)	4 (0.10)	9 (0.15)			
**HDL-C ranges (mg/dL), n (%)**							767.1 (4)	<.001
	<35		236 (12.18)	1553 (38.70)	1789 (30.06)			
	35-44		378 (19.50)	1034 (25.77)	1412 (23.73)			
	45-49		323 (16.67)	544 (13.56)	867 (14.57)			
	50-59		541 (27.92)	629 (15.67)	1170 (19.66)			
	>59		460 (23.74)	253 (6.3)	713 (11.98)			
	**Self-reported**						429.4 (4)	<.001
		<35	331 (17.08)	1603 (39.95)	1934 (32.50)			
		35-44	514 (26.52)	1112 (27.71)	1626 (27.32)			
		45-49	501 (25.85)	693 (17.27)	1194 (20.06)			
		50-59	381 (19.66)	448 (11.16)	829 (13.93)			
		>59	211 (10.89)	157 (3.91)	368 (6.18)			
**LDL-C ranges (mg/dL), n (%)**							3.8 (4)	.44
	<100		645 (33.28)	1361 (33.91)	2006 (33.71)			
	100-129		631 (32.56)	1349 (33.62)	1980 (33.27)			
	130-159		439 (22.65)	895 (22.30)	1334 (22.42)			
	160-189		162 (8.36)	310 (7.72)	472 (7.93)			
	>189		61 (3.15)	98 (2.44)	159 (2.67)			
	**Self-reported**						11.9 (4)	.02
		<100	497 (25.64)	1178 (29.35)	1675 (28.15)			
		100-129	639 (32.97)	1323 (32.97)	1962 (32.97)			
		130-159	544 (30.03)	1013 (25.24)	1557 (26.16)			
		160-189	181 (9.34)	365 (9.10)	546 (9.17)			
		>189	77 (3.97)	134 (3.34)	211 (3.55)			
**Total cholesterol ranges, (mg/dL), n (%)**							81.8 (4)	<.001
	<160		489 (25.23)	695 (17.32)	1184 (19.90)			
	160-199		762 (39.32)	1464 (36.48)	2226 (37.41)			
	200-239		497 (25.64)	1307 (32.57)	1804 (30.31)			
	240-279		143 (7.38)	438 (10.91)	581 (9.76)			
	>279		47 (2.43)	109 (2.72)	156 (2.62)			
	**Self-reported**						53.7 (4)	<.001
		<160	420 (21.67)	729 (18.17)	1149 (19.31)			
		160-199	763 (39.37)	1351 (33.67)	2114 (35.52)			
		200-239	582 (30.03)	1384 (34.49)	1996 (33.04)			
		240-279	130 (6.71)	436 (10.86)	566 (9.51)			
		>279	43 (2.22)	113 (2.82)	156 (2.62)			

^a^Bonferroni correction was used to determine statistical significance based on 14 comparisons (alpha=.004).

**Table 5:** Differences in means and proportions by sex in the pairwise deleted sample.

Variables in dataset			N	Male	Female	Total	*t* (*df*)	χ^2^(*df*)	*P* ^*a*^
Age, mean (SD)			18,428	53.7 (12.0)	54.1. (11.5)	54.0 (11.7)	–2.0 (18,426)		.004
**Race/ethnicity, n (%)**			18,659	5529	13,130	18,659		70.6 (5)	<.001
	Non-Hispanic white			4612 (83.41)	10,729 (81.71)	15,341 (82.22)			
	African-American			426 (7.70)	1417 (10.79)	1843 (9.88)			
	Hispanic			263 (4.76)	610 (4.65)	873 (4.68)			
	Asian/Pacific Islander			140 (2.53)	185 (1.41)	325 (1.74)			
	American Indian/Alaskan Native			13 (0.24)	44 (0.34)	57 (0.31)			
	Other			75 (1.36)	145 (1.10)	220 (1.18)			
**Tobacco use, n (%)**			18,724	5549	13175	18,724		53.7 (2)	<.001
	Never			3545 (63.89)	9138 (69.36)	12683 (67.74)			
	Quit			1320 (23.79)	2679 (20.33)	3999 (21.36)			
	Yes			684 (12.33)	1358 (10.31)	2042 (10.91)			
**Diabetes, n (%)**			18,724	5549	13,175	18,724		6.7 (4)	.15
	No			5049 (90.99)	12,104 (91.87)	17,153 (91.61)			
	Type 1			32 (0.58)	72 (0.55)	104 (0.56)			
	Type 2			420 (7.57)	889 (6.75)	1309 (6.99)			
	Borderline			15 (0.27)	49 (0.37)	64 (0.34)			
	Unsure			33 (0.59)	61 (0.46)	94 (0.50)			
**Systolic blood pressure ranges (mm Hg), n (%)**			18,270	5407	12,863	18,270		210.9 (5)	<.001
	<120			1283 (23.73)	4350 (33.82)	5633 (30.83)			
	120-129			1466 (27.11)	3416 (26.56)	4882 (26.72)			
	130-139			1221 (22.58)	2270 (17.65)	3491 (19.11)			
	140-159			1141 (22.10)	2268 (17.63)	3409 (18.66)			
	160-199			284 (5.25)	544 (4.23)	828 (4.53)			
	>199			12 (0.22)	15 (0.12)	27 (0.15)			
	**Self-reported**		16,171	4710	11,461	16,171		274.8 (5)	<.001
		<120		838 (17.79)	3339 (29.13)	4177 (25.83)			
		120-129		1706 (36.22)	4097 (35.75)	5803 (35.89)			
		130-139		1250 (26.54)	2253 (19.66)	3503 (21.66)			
		140-159		737 (15.65)	1469 (12.82)	2206 (13.64)			
		160-199		169 (3.59)	291 (2.54)	460 (2.84)			
		>199		10 (0.21)	12 (0.10)	22 (0.14)			
**Diastolic blood pressure ranges (mm Hg), n (%)**			18,202	5392	12,810	18,202		338.2 (5)	<.001
	<80			2161 (40.08)	6833 (53.34)	8994 (49.41)			
	80-84			1511 (28.02)	3275 (25.57)	4786 (26.29)			
	85-89			701 (13.0)	1184 (9.24)	1885 (10.36)			
	90-99			819 (15.19)	1252 (9.77)	2071 (11.38)			
	100-114			178 (3.30)	251 (1.96)	429 (2.36)			
	>114			22 (0.41)	15 (0.12)	37 (0.20)			
	**Self-reported**		16,013	4685	11,328	16,013		216.5 (5)	<.001
		<80		1611 (34.39)	5167 (45.61)	6778 (42.33)			
		80-84		1576 (33.64)	3581 (31.61)	5157 (32.21)			
		85-89		834 (17.80)	1467 (12.95)	2301 (14.37)			
		90-99		536 (11.44)	907 (8.01)	1443 (9.01)			
		100-114		104 (2.2)	180 (1.59)	284 (1.77)			
		>114		24 (0.51)	26 (0.23)	50 (0.31)			
**HDL-C ranges (mg/dL), n (%)**			16,779	5048	11,731	16,779		2,200.0 (4)	<.001
	<35			539 (10.68)	4162 (35.48)	4701 (28.02)			
	35-44			829 (16.42)	2947 (25.12)	3776 (22.50)			
	45-49			680 (13.47)	1581 (13.48)	2261 (13.48)			
	50-59			1575 (31.20)	2129 (18.15)	3704 (22.8)			
	>59			1425 (28.23)	912 (7.7)	2337 (13.93)			
	**Self-reported**		7691	2518	5173	7691		617.5 (4)	<.001
		<35		399 (15.85)	2022 (15.3)	2421 (31.48)			
		35-44		627 (24.90)	1409 (10.7)	2036 (26.47)			
		45-49		626 (24.86)	873 (6.6)	1499 (19.49)			
		50-59		509 (20.21)	622 (4.7)	1131 (14.71)			
		>59		357 (14.18)	247 (4.77)	604 (7.85)			
**LDL-C ranges (mg/dL), n (%)**			14,476	4370	10106	14,476		16.8 (4)	<0.001
	<100			1336 (30.57)	3334 (32.99)	4670 (32.26)			
	100-129			1476 (33.78)	3455 (34.19)	4931 (34.06)			
	130-159			1006 (23.02)	2227 (22.04)	3233 (22.33)			
	160-189			396 (9.06)	801 (7.93)	1197 (8.27)			
	>189			156 (3.57)	289 (2.86)	445 (3.07)			
	**Self-reported**		6769	2196	4573	6769		12.9 (4)	.01
		<100		553 (25.18)	1312 (28.69)	1865 (27.55)			
		100-129		717 (32.65)	1517 (33.17)	2234 (33.00)			
		130-159		610 (27.78)	1141 (24.95)	1751 (25.87)			
		160-189		218 (9.93)	418 (9.14)	636 (9.40)			
		>189		98 (4.46)	185 (4.05)	283 (4.18)			
**Total cholesterol ranges (mg/dL), n (%)**			17,627	5265	12,362	17,627		137.9 (4)	<.001
	<160			1128 (21.42)	1901 (15.38)	3029 (17.18)			
	160-199			2050 (38.94)	4628 (37.44)	6678 (37.89)			
	200-239			1467 (27.86)	4100 (33.17)	5567 (31.58)			
	240-279			450 (8.55)	1371 (11.09)	1821 (10.33)			
	>279			170 (3.23)	362 (2.93)	532 (3.02)			
	**Self-reported**		11,541	3495	8046	11,541		49.8 (4)	<.001
		<160		661 (18.91)	1281 (15.92)	1942 (16.83)			
		160-199		1292 (36.97)	2687 (33.40)	3979 (34.48)			
		200-239		1139 (32.59)	2900 (36.04)	4039 (35.00)			
		240-279		291 (8.33)	898 (11.16)	1189 (10.30)			
		>279		112 (3.20)	280 (3.48)	392 (3.40)			

^a^Bonferroni correction was used to determine statistical significance based on 14 comparisons (alpha=.004).

**Table 6:** Differences in means and proportions by sex for imputed sample (n=16,879).

Variables in dataset			Male (n=5011)	Female (n=11,868)	Total (n=16,879)	*t* (*df*)	χ^2^(*df*)	*P* ^a^
Age, mean (SD)			53.7 (12.1)	54.2 (11.5)	54.0 (11.7)	2.4 (16,877)		.01
**Race/ethnicity, n (%)**							70.5 (5)	<.001
	Non-Hispanic white		4168 (83.18)	9648 (81.29)	13,816 (81.85)			
	African-American		385 (7.68)	1321 (11.13)	1706 (10.11)			
	Hispanic		245 (4.89)	552 (4.65)	797 (4.72)			
	Asian/Pacific Islander		119 (2.37)	161 (1.36)	280 (1.66)			
	American Indian/ Alaskan Native		12 (0.24)	43 (0.36)	55 (0.33)			
	Other		82 (1.64)	143 (1.20)	225 (1.33)			
**Tobacco use, n (%)**							56.7 (2)	<.001
	Never		3190 (63.66)	8257 (69.57)	11,447 (67.82)			
	Quit		1220 (24.35)	2398 (20.21)	3618 (21.43)			
	Yes		601 (11.99)	1213 (10.22)	1814 (10.75)			
**Diabetes, n (%)**							5.4 (4)	.25
	No		4557 (90.94)	10883 (91.70)	15,440 (91.47)			
	Type 1		27 (0.54)	60 (0.51)	87 (0.52)			
	Type 2		383 (7.64)	826 (6.96)	1209 (7.16)			
	Borderline		14 (0.28)	46 (0.39)	60 (0.36)			
	Unsure		30 (0.60)	53 (0.45)	83 (0.49)			
**Systolic blood pressure ranges (mm Hg), n (%)**							182.7 (5)	<.001
	<120		1220 (24.35)	4049 (34.12)	5269 (31.22)			
	120-129		1352 (26.98)	3131 (26.38)	4483 (26.56)			
	130-139		1130 (22.55)	2096 (17.66)	3226 (19.11)			
	140-159		1043 (20.81)	2087 (17.59)	3130 (18.54)			
	160-199		255 (5.09)	493 (4.15)	748 (4.43)			
	>199		11 (0.22)	12 (0.10)	23 (0.14)			
	**Self-reported**						226.4 (5)	<.001
		<120	931 (18.58)	3428 (28.88)	4359 (25.82)			
		120-129	1833 (36.58)	4204 (35.42)	6037 (35.77)			
		130-139	1288 (25.70)	2368 (19.95)	3656 (21.66)			
		140-159	772 (15.41)	1530 (12.89)	2302 (13.64)			
		160-199	176 (3.51)	325 (2.74)	501 (2.97)			
		>199	11 (0.22)	13 (0.11)	24 (0.14)			
**Diastolic blood pressure ranges (mm Hg), n (%)**							294.3 (5)	<.001
	<80		2042 (40.75)	6370 (53.67)	8412 (49.84)			
	80-84		1401 (27.96)	3008 (25.35)	4409 (26.12)			
	85-89		640 (12.77)	1087 (9.16)	1727 (10.23)			
	90-99		742 (14.81)	1154 (9.72)	1896 (11.23)			
	100-114		165 (3.29)	235 (1.98)	400 (2.37)			
	>114		21 (0.42)	14 (0.12)	35 (0.21)			
	**Self-reported**						172.3 (5)	<.001
		<80	1784 (35.60)	5376 (45.30)	7160 (42.42)			
		80-84	1665 (33.23)	3722 (31.36)	5387 (31.92)			
		85-89	875 (17.46)	1568 (13.21)	2443 (14.47)			
		90-99	546 (10.90)	987 (8.32)	1533 (9.08)			
		100-114	114 (2.27)	189 (1.59)	303 (1.80)			
		>114	27 (0.54)	26 (0.22)	53 (0.31)			
**HDL-C ranges (mg/dL), n (%)**							1800.0 (4)	<.001
	<35		594 (11.85)	4100 (34.55)	4694 (27.81)			
	35-44		844 (16.84)	2947 (24.83)	3791 (22.46)			
	45-49		672 (13.41)	1568 (13.21)	2240 (13.27)			
	50-59		1543 (30.79)	2248 (18.94)	3791 (22.46)			
	>59		1358 (27.10)	1005 (8.47)	2363 (14.00)			
	**Self-reported**						295.9 (4)	<.001
		<35	1101 (21.97)	3994 (33.65)	5095 (30.19)			
		35-44	1262 (25.18)	3085 (25.99)	4347 (25.75)			
		45-49	1148 (22.91)	2180 (18.37)	3328 (19.72)			
		50-59	913 (18.22)	1693 (14.27)	2606 (15.44)			
		>59	587 (11.71)	916 (7.72)	1503 (8.90)			
**LDL-C ranges (mg/dL), n (%)**							9.7 (4)	0.046
	<100		1554 (31.01)	3935 (33.16)	5489 (32.52)			
	100-129		1713 (34.18)	4007 (33.76)	5720 (33.89)			
	130-159		1137 (22.69)	2621 (22.08)	3758 (22.26)			
	160-189		444 (8.86)	962 (8.11)	1406 (8.33)			
	>189		163 (3.25)	343 (2.89)	506 (3.00)			
	**Self-reported**						9.8 (4)	.04
		<100	1296 (25.86)	3307 (27.86)	4603 (27.27)			
		100-129	1656 (33.05)	3922 (33.05)	5578 (33.05)			
		130-159	1357 (27.08)	3005 (25.32)	4362 (25.84)			
		160-189	470 (9.38)	1122 (9.45)	1582 (9.43)			
		>189	232 (4.63)	522 (4.40)	754 (4.47)			
**Total cholesterol ranges (mg/dL), n (%)**							130.8 (4)	<.001
	<160		1085 (21.65)	1852 (15.60)	2937 (17.40)			
	160-199		1941 (38.73)	4421 (37.25)	6362 (37.69)			
	200-239		1389 (27.72)	3920 (33.03)	5309 (31.45)			
	240-279		434 (8.66)	1330 (11.21)	1764 (10.45)			
	>279		162 (3.23)	345 (2.91)	507 (3.00)			
	**Self-reported**						28.1 (4)	<.001
		<160	938 (18.72)	2002 (16.87)	2940 (17.42)			
		160-199	1787 (35.66)	3972 (33.47)	5759 (34.12)			
		200-239	1657 (33.07)	4150 (34.97)	5807 (34.40)			
		240-279	469 (9.36)	1336 (11.26)	1805 (10.69)			
		>279	160 (3.19)	408 (3.44)	568 (3.37)			

^a^Bonferroni correction was used to determine statistical significance based on 14 comparisons (alpha=.004)

### Weighted Kappa Agreement and Agreement by Risk Stratification

#### Listwise Deletion

Table 7 reports the results of the weighted kappa interrater agreement analysis by sex for each CHD risk factor variable in the listwise deleted sample. In addition to the weighted kappa statistic, its standard error and bias-corrected 95% confidence interval was reported along with an estimate of the 10-year CHD risk score for each variable’s strata of clinical values.

**Table 7:** Interrater agreement of self-reported and clinically measured Framingham 10-year CHD risk factors by risk score for the listwise deleted sample (n=5951).

Clinically measured risk factor	Males (n=1938)					Females (n=4013)				
	Risk score^a^	n	Weighted kappa statistics			Risk score^a^	n	Weighted kappa statistics		
			κ	SE	95% CI^b^			κ	SE	95% CI^b^
**Total cholesterol**		1938	.56	.01	.53-.59	4013	4013	.59	.01	.57-.61
	<3%	518	.52	.03	.46-.57	1123	1123	.64	.02	.60-.67
	3-4%	538	.59	.02	.53-.62	1157	1157	.53	.02	.49-.56
	5-6%	524	.54	.03	.48-.60	1178	1178	.55	.02	.51-.59
	>6%	358	.55	.03	.50-.62	555	555	.53	.03	.48-.59
**HDL-C**		1938	.49	.02	.46-.52	4013	4013	.57	.01	.56-.60
	<3%	518	.56	.03	.50-.60	1123	1123	.60	.02	.57-.64
	3-4%	538	.54	.02	.49-.58	1157	1157	.55	.02	.53-.59
	5-6%	524	.44	.03	.38-.48	1178	1178	.58	.02	.54-.61
	>6%	358	.33	.03	.25-.37	555	555	.45	.03	.41-.50
**LDL-C**		1938	.58	.02	.55-.62	4013	4013	.58	.01	.55-.60
	<3%	518	.48	.03	.43-.54	1123	1123	.60	.02	.57-.65
	3-4%	538	.58	.03	.52-.63	1157	1157	.49	.02	.45-.53
	5-6%	524	.60	.03	.53-.67	1178	1178	.55	.02	.51-.59
	>6%	358	.57	.03	.50-.63	555	555	.52	.03	.48-.58
**Systolic blood pressure**		1938	.42	.01	.38-.44	4013	4013	.45	.01	.42-.47
	<3%	518	.47	.03	.40-.52	1123	1123	.51	.02	.48-.56
	3-4%	538	.44	.03	.37-.49	1157	1157	.42	.02	.39-.46
	5-6%	524	.39	.03	.33-.47	1178	1178	.40	.02	.35-.43
	>6%	358	.34	.04	.28-.41	555	555	.36	.03	.31-.43
**Diastolic blood pressure**		1938	.42	.01	.40-.45	4013	4013	.43	.01	.43-.46
	<3%	518	.49	.03	.43-.55	1123	1123	.50	.02	.46-.54
	3-4%	538	.41	.03	.35-.47	1157	1157	.42	.02	.38-.46
	5-6%	524	.38	.03	.31-.43	1178	1178	.38	.02	.33-.43
	>6%	358	.40	.04	.33-.47	555	555	.38	.03	.31-.43
**Tobacco user** ^c^		165				264				
	≤5%	79				156				
	>5%	86				108				
**Diagnosed with diabetes** ^c^		200				345				
	≤5%	95				179				
	>5%	105				166				

^a^Framingham 10-year CHD risk score.

^b^Bias-corrected 95% CI.

^c^Interrater agreement not measured.

#### Pairwise Deletion

Table 8 reports the results of the weighted kappa interrater agreement analysis by sex for each CHD risk factor variable in the pairwise deleted sample. In addition to the weighted kappa statistic, its standard error and bias-corrected 95% confidence interval was reported along with an estimate of the 10-year CHD risk score for each variable’s strata of clinical values.

**Table 8:** Interrater agreement of self-reported and clinically measured Framingham 10-year CHD risk factors by risk score for the pairwise deleted sample.

Clinically measured risk factor	Males (n=varies)					Females (n=varies)				
	Risk score^a^	n	Weighted kappa statistics			Risk score^a^	n	Weighted kappa statistics		
			κ	SE	95% CI^b^			κ	SE	95% CI^b^
**Total cholesterol**		4524	.61	.01	.59-.63		9547	.58	.01	.57-.60
	<3%	1038	.57	.02	.53-.61	<4%	2410	.62	.01	.59-.63
	3-4%	1176	.61	.02	.57-.64	4-6%	2523	.52	.01	.50-.55
	5-6%	1168	.59	.02	.56-.62	7-8%	3009	.57	.01	.55-.59
	>6%	1142	.64	.02	.61-.69	>8%	1605	.53	.02	.53-.60
**HDL-C**		3374	.58	.01	.55-.60		6217	.64	.01	.63-.65
	<3%	773	.63	.02	.58-.68	<4%	1600	.65	.01	.62-.68
	3-4%	861	.62	.02	.58-.66	4-6%	1649	.60	.02	.56-.63
	5-6%	893	.54	.02	.50-.57	7-8%	1948	.65	.01	.63-.69
	>6%	847	.47	.03	.43-.52	>8%	1020	.57	.02	.54-.61
**LDL-C**		2624	.58	.01	.56-.60		4989	.58	.01	.56-.60
	<3%	627	.49	.03	.44-.53	<4%	1318	.58	.02	.54-.61
	3-4%	707	.59	.02	.54-.64	4-6%	1441	.50	.02	.47-.53
	5-6%	697	.61	.03	.56-.66	7-8%	1497	.55	.02	.51-.58
	>6%	593	.55	.03	.49-.60	>8%	733	.54	.02	.50-.58
**Systolic blood pressure**		5951	.47	.01	.45-.49		13,381	.47	.01	.46-.48
	<3%	1439	.46	.02	.42-.50	<4%	3744	.49	.01	.46-.50
	3-4%	1552	.45	.02	.42-.49	4-6%	3432	.41	.01	.39-.43
	5-6%	1472	.44	.02	.42-.47	7-8%	3967	.44	.01	.43-.46
	>6%	1488	.46	.02	.43-.50	>8%	2238	.43	.01	.40-.45
**Diastolic blood pressure**		5901	.47	.01	.45-.49		13,199	.43	.01	.42-.45
	<3%	1430	.44	.02	.41-.49	<4%	3709	.46	.01	.44-.48
	3-4%	1539	.45	.02	.42-.49	4-6%	3385	.40	.01	.38-.44
	5-6%	1464	.46	.02	.43-.50	7-8%	3905	.42	.01	.40-.44
	>6%	1468	.51	.02	.48-.54	>8%	2200	.43	.02	.40-.45
**Tobacco user** ^c^		876					1591			
	≤5%	409				≤7%	887			
	>5%	467				>7%	704			
**Diagnosed with diabetes** ^c^		693					1256			
	≤5%	224				≤7%	511			
	>5%	469				>7%	745			

^a^Framingham 10-year CHD risk score.

^b^Bias-corrected 95% CI.

^c^Interrater agreement not measured.

#### Imputation

Table 9 reports the results of the weighted kappa interrater agreement analysis by sex for each CHD risk factor variable in the imputed sample. In addition to the weighted kappa statistic, its standard error and bias-corrected 95% confidence interval was reported along with an estimate of the 10-year CHD risk score for each variable’s strata of clinical values.

**Table 9:** Imputed sample (n=16,879): interrater agreement of self-reported and clinically measured Framingham 10-year CHD risk factors by risk score.

Clinically measured risk factor	Males (n=5011)					Females (n=11,868)				
	Risk score^a^	n	Weighted kappa statistics			Risk score^a^	n	Weighted kappa statistics		
			κ	SE	95% CI^b^			κ	SE	95% CI^b^
**Total cholesterol**		5011	.36	.01	.34-.38		11,868	.35	.01	.34-.37
	<3%	1406	.30	.02	.26-.33	<3%	2902	.34	.01	.31-.36
	3-4%	1288	.38	.02	.35-.43	3-5%	3071	.36	.01	.33-.38
	5-6%	1207	.37	.02	.33-.41	6-8%	3237	.34	.01	.32-.38
	>6%	1110	.37	.02	.34-.44	>8%	2658	.33	.01	.30-.36
**HDL-C**		5011	.25	.01	.23-.27		11,868	.28	.01	.27-.29
	<3%	1406	.28	.02	.25-.31	<3%	2902	.28	.01	.25-.30
	3-4%	1288	.27	.02	.23-.30	3-5%	3071	.36	.01	.33-.38
	5-6%	1207	.24	.02	.21-.27	6-8%	3237	.27	.01	.25-.30
	>6%	1110	.18	.02	.15-.21	>8%	2658	.20	.01	.18-.22
**LDL-C**		5011	.24	.01	.22-.27		11,868	.21	.01	.20-.23
	<3%	1406	.18	.02	.14-.21	<3%	2902	.19	.01	.16-.21
	3-4%	1288	.25	.02	.21-.28	3-5%	3071	.23	.01	.20-.25
	5-6%	1207	.29	.02	.26-.33	6-8%	3237	.21	.01	.19-.24
	>6%	1110	.25	.02	.19-.28	>8%	2658	.22	.01	.19-.23
**Systolic blood pressure**		5011	.38	.01	.36-.41		11,868	.39	.01	.37-.40
	<3%	1406	.38	.02	.34-.41	<3%	2902	.33	.01	.30-.35
	3-4%	1288	.35	.02	.31-.39	3-5%	3071	.34	.01	.31-.36
	5-6%	1207	.35	.02	.31-.39	6-8%	3237	.31	.01	.29-.33
	>6%	1110	.33	.02	.31-.37	>8%	2658	.30	.01	.28-.33
**Diastolic blood pressure**		5011	.37	.01	.34-.39		11,868	.35	.01	.33-.36
	<3%	1406	.35	.02	.32-.38	<3%	2902	.34	.02	.32-.38
	3-4%	1288	.37	.02	.32-.41	3-5%	3071	.35	.01	.33-.38
	5-6%	1207	.37	.02	.32-.41	6-8%	3237	.33	.02	.30-.36
	>6%	1110	.38	.02	.34-.42	>8%	2658	.31	.01	.28-.33
**Tobacco user** ^c^		601					1213			
	≤5%	295				≤6%	629			
	>5%	306				>6%	584			
**Diagnosed with diabetes** ^c^		410					886			
	<5%	153				≤6%	279			
	>5%	257				>6%	607			

^a^Framingham 10-year CHD risk score.

^b^Bias-corrected 95% CI.

^c^Interrater agreement not measured.

When evaluating the trends of interrater agreement between self-reported and clinically measured CHD risk factors, it is important to evaluate both the baseline interrater agreement coefficients for the entire sample and the individual interrater agreement coefficients for the strata based on 10-year CHD risk. Further, it is important to examine the changes in the interrater agreement as 10-year CHD risk increases.

Although there are some noteworthy differences between the listwise deleted and pairwise deleted samples (eg, the deterioration of interrater agreement by strata for SBP and DBP as 10-year CHD risk increases among males in the listwise deleted sample but not the pairwise deleted sample), the main outcome of interest is the difference in baseline interrater agreement coefficients of the imputed sample versus the listwise and pairwise deleted samples. Overall, the baseline interrater agreement coefficient values for each risk factor in the imputed sample were markedly lower than their counterparts in the listwise and pairwise deleted samples. For example, among males in the listwise deleted sample, the interrater agreement coefficient of self-reported and clinically measured ranges of HDL-C was kappa=.49. By comparison, the same coefficient in the imputed sample was .25. This discrepancy was substantial across the risk factor values with the largest amount of missing data (ie, ranges of TC, HDL-C, and LDL-C). By comparison, the differences in interrater agreement coefficients of variables other than ranges of TC, HDL-C, and LDL-C between the listwise deleted and imputed samples were minor. For example, among females in the listwise deleted sample, the interrater agreement coefficient of self-reported and clinically measured SBP was .45. By comparison, the same coefficient in the imputed sample was .39.

As discussed previously, one of the CHD risk factors thought to be less understood by community-dwelling adults is HDL-C. It is noteworthy that both males and females with the highest 10-year risk of CHD in the imputation sample had the lowest level of interrater agreement between self-reported and clinically measured ranges of HDL-C. In fact, the level of agreement can only be characterized as slight, which is a suboptimal level of agreement. Although the difference between a 3% 10-year risk of CHD and an 8% 10-year risk of CHD may seem numerically immaterial, it should be noted these figures are derived from Framingham’s clinical risk model [24], which means the difference between 3% and 8% is more than twice the mortality risk of CHD in the next 10 years. Thus, the difference is clinically relevant.

Conversely, interrater agreement of self-reported and clinically measured ranges of LDL-C slightly increased in both sexes as 10-year CHD risk increased. This is consistent with the layperson hypothesis that individuals with higher risk of CHD would be more conscious of LDL-C because it is often referred to as “bad” cholesterol. This finding is also supported with recent evidence suggesting diabetes patients who recall their most recent LDL-C values are more likely to maintain optimal hemoglobin A1C values [25]. LDL-C could simply be the metric noted by community-dwelling adults as the most important metric to gauge in order to avoid CHD and related diseases. This is certainly consistent with how patients have been conditioned to assume LDL-C is bad cholesterol and HDL-C is good cholesterol (a belief that is the subject of rigorous investigation) [26]. If HDL-C is eventually deemed to be just as clinically important as LDL-C, a substantial public health information campaign may be necessary to inculcate this knowledge and its importance among a public much more likely to appreciate CHD risk due to LDL-C.

### Sensitivity Missing Data Techniques

Upon examining the differences of interrater agreement coefficients by the approach used to address missing data, 2 things become apparent. First, ranges of SBP, DBP, and both tobacco use and DM status were not substantially different based on the approach employed to account for missing data. This was mostly because of fewer instances of missing data than other variables in the original dataset. As such, it is appropriate to use any of the 3 samples to establish findings about interrater agreement relative to these variables in the study. However, given the significant amount of missing data for ranges of TC, HDL-C, and LDL-C, the multiple imputation strategy resulted in more conservative results of interrater agreement than the listwise and pairwise deleted samples. As such, the researcher is cautioned to use these figures when establishing findings from the study. Because of these facts, the multiple imputation sample was deemed the most appropriate for discussing findings of this study. This is because the imputation sample was conservative on the variables with greatest instances of missing data, but consistent with the other 2 methodologies for the variables with fewer instances of missing data.

### Sample Versus Population-Based Coronary Heart Disease Risk Data

**Figure 1:**
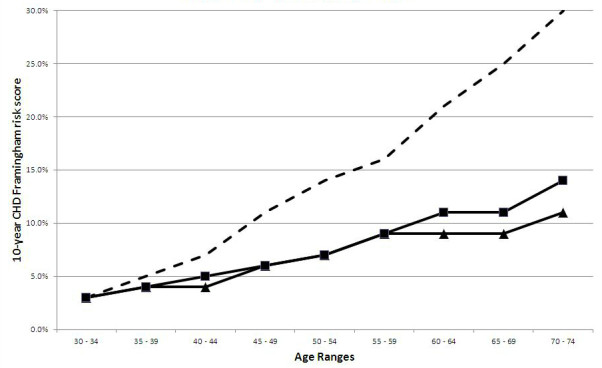
Comparison of CHD risk by sample type relative to the overall population for males.

**Figure 2:**
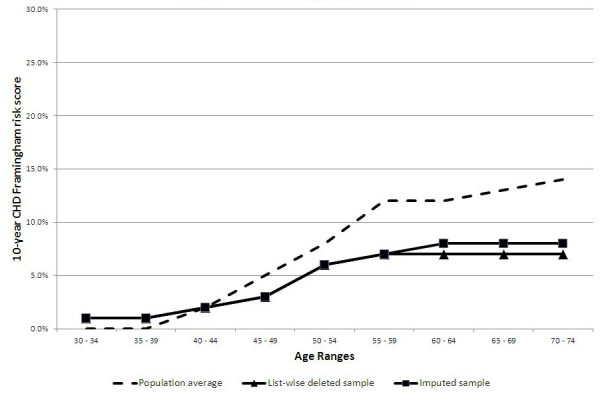
Comparison of CHD risk by sample type relative to the overall population for females.

Figure 1 illustrates the comparative 10-year CHD risk score as established by the Framingham Heart Study [24] for the general male population by age group. The results from the listwise deleted and imputed samples, respectively, are also shown for comparison. Figure 2 illustrates the comparative 10-year CHD risk score as established by the Framingham Heart Study [24] for the general female population by age group. The results from the listwise deleted and imputed samples, respectively, are also shown for comparison.

## Discussion

### Heart Aware May Underestimate Population Risk for Coronary Heart Disease

The most significant finding from this study is the fact a community-based health risk assessment for heart disease that is delivered via the Internet (Heart Aware) yields a sample with markedly lower risk of CHD than suggested by population health data. For example, Figure 1 demonstrates that despite the method used to account for missing data, those males participating in the Heart Aware assessment had, on average, a 10-year CHD risk that was up to 19 percentage points lower than their counterparts of the same age. Likewise, Figure 2 demonstrates that despite the method used to account for missing data, those females participating in the Health Aware assessment had, on average, a 10-year CHD risk that was up to 7 percentage points lower than their counterparts of the same age.

It should be noted this problem is compounded by the fact participants selected for clinical evaluation in this study were hand-picked by the individual hospitals based on their perceived high risk of CHD (ie, a random selection of community-dwelling adults for clinical measurement of CHD risk factors would likely result in samples with lower CHD risk, thereby exacerbating the differences between the risk of samples established by the Heart Aware assessment versus population health data), recognizing that anyone with diagnosed CHD was excluded from the study.

There are several reasons that could explain these discrepancies. The Heart Aware assessments were offered almost entirely via the Internet. This probably resulted in biased selection of participants because those using the Internet are generally more technically savvy, have higher levels of education and income, and are comparatively healthier than non-Internet users [27]. As such, it is not surprising the tool procured a lower-risk population that is not representative of the general population. This raises a very important issue about health risk assessments such as Heart Aware. Obviously, recruitment cost is greatly reduced by using the Internet, especially through a hospital’s existing Internet presence. However, if the data are to be used for public health purposes, how can the data be more representative of the population? One approach could be to expand the methods used to collect the same data, such as using in-clinic kiosks to collect data versus relying on a participant having Internet resources available at home. If financial resources were available, the instrument could be made available through a random-digit dial survey. This method has been shown to improve validity in other CVD-related studies [28]. Finally, a simple and cost-effective method could be to use propensity score matching to create appropriate comparison groups for analysis. This approach is also common in CVD-related studies [29]. However, in the case of the current Heart Aware survey, additional variables need to be collected to account for the underlying demographic differences that are likely associated with the Internet selection bias discussed previously (eg, income, education level, and current health care utilization).

It is difficult to understand the influence of the selection issue on the study results. Although we know the samples in this study were of individuals of much lower CHD risk relative to population health data, and we did exclude those who had already been diagnosed with CHD, the relationship between levels of CHD risk and knowledge of CHD risk factors has yet to be firmly established. In fact, in this study, differences in the interrater agreement of self-reported and clinically measured CHD risk factors varied by sex, individual risk factor, and overall 10-year CHD risk stratum.

### Sex-Based Differences Were Not Apparent

As noted previously, the Red Dress symbol and Go Red for Women campaign have been high profile efforts to highlight the fact more females of all ages die of CHD than any other cause of death [30]. Yet, the results of this study indicate a very similar level of awareness of CHD risk factors among the sexes. It is difficult to reconcile this data with the potential success or failure of these high profile campaigns specific to females. Although there may be no marked difference in the interrater agreement between males and females on each measured CHD risk factor in the imputed sample, perhaps awareness would have been worse without the public campaigns focused on females?

What is clear about the differences in the interrater agreement of self-reported and clinically measured CHD risk factors by sex is neither sex has demonstrated a superior understanding of their CHD risk factors. Both sexes demonstrate relatively low levels of agreement on every CHD risk factor. It should also be noted females were generally “healthier” than their male counterparts in this study (see Table 6). It seems rational such a difference would influence CHD risk factor agreement by sex to a greater degree than witnessed in this study. This is an area for continued exploration because it is central to public health policy.

### Limitations

In addition to the noteworthy findings of this study, there are several limitations. First, the most substantial limitation is a challenge to internal validity of the results based on a substantial amount of selection bias that was likely the result of the recruitment method (ie, offering the survey to any interested party through media advertising). However, as Guba [31] reminds us in a classic work on naturalistic studies, the process of determining validity is not comparable with rationalistic designs such as randomized controlled trials. Naturalistic trials have a wide array of tools to complement the rationalistic approach to establishing comparable levels of study integrity and quality [31]. Among these methods are techniques such as triangulation of results, replication, and comprehensive descriptive statistics to ensure a thorough understanding of the sample [31]. If naturalistic designs are fundamentally characterized as research conducted in natural settings versus structured environments such as laboratories [32], then Heart Aware should qualify as a tool used in a naturalistic setting. Because of this paradigm, the study does accomplish some of the processes desired by naturalistic researchers, such as the exhaustive approach to examining missing data, the use of multiple imputation to ensure replication of the results displayed in the imputed sample, and the 100 repetitions conducted on each weighted kappa analysis of interrater agreement between self-reported and clinically measured CHD risk factors. Nevertheless, future research in this area should incorporate some of the suggestions made previously to counteract the apparent selection bias of solely using the Internet for recruitment.

The second limitation is the amount of missing data. Although this study has attempted to mitigate this point with multiple approaches, none of these efforts can fully account for the bias that exists in statistical estimation as a result of missing responses. At a very basic level, the latent traits of missing responses remain unknown even with the most sophisticated missing data techniques. However, it should be noted repetition and replication (as noted previously) somewhat mitigate these biases.

The third limitation of the study is the difference between sexes for baseline health behaviors and clinical values. Although some of the clinical value differences are because of normal differences based on sex, some of the discrepancies are very large indicating females are probably healthier than their male counterparts. This influences the ability to fully understand results of the study by sex.

The fourth limitation of the study is the lack of research on how respondents acquired information about their self-reported risk factor values. There could be an element of self-education or access to professional resources that play a role in the findings of the study.

Finally, it was not possible to exclude individuals from the study who had undiagnosed CHD. The sample likely contained some of these individuals and could have contributed to selection bias concerns.

### Future Research

The findings from this study have a unique place in the literature based on the large sample size, breadth of heart disease self-reported risk factors collected, and the method of data collection (ie, the Internet). However, this was a cross-sectional study that lacks the internal validity of a stronger design such as a randomized controlled trial. Future efforts in this field would benefit from a prospective randomized study design to ensure some of the self-selection biases and other limitations of this study are appropriately addressed.

### Conclusions

This study sought to understand which CHD risk factors were best understood by community-dwelling adults who took an Internet-based CHD risk assessment (ie, Heart Aware). It also sought to examine whether such levels of understanding were associated with varying degrees of 10-year CHD risk for each participant. What the study has shown is although all CHD risk factors had suboptimal levels of interrater agreement between self-reported and clinically measured values, the CHD risk factor with the greatest discordance was HDL-C. This is consistent with the literature noted previously. However, this study provides unique support to this finding by incorporating a thorough review of how interrater agreement coefficients change based on approaches to missing data. Because missing data are a key analytical issue in many surveillance studies [33], the current study provides a robust view that supports the findings of interrater agreement for HDL-C in a variety of methodological settings. Further, these findings were drawn from a very large sample across more than 100 hospitals.

Unlike prior research efforts, this study stratified interrater agreement of self-reported and clinically measured CHD risk factors by 10-year CHD risk as established by the Framingham Heart Study [24]. This allowed the current study to make a very important contribution to the literature, the discovery that interrater agreement for HDL-C deteriorates as 10-year CHD risk increases, whereas interrater agreement for LDL-C improves as 10-year CHD risk increases. This is a powerful finding because it not only supports the literature noted previously regarding the lack of knowledge of HDL-C among community-dwelling adults, but it also shows how the same individuals also view LDL-C. This finding has substantial implications for the health literacy, social and behavioral health, and public health implementation science communities. If the evidence of HDL-C as a protective factor for CHD continues to mature, it will be vital to translate these clinical findings into actionable public health information campaigns in the community.

Several broad themes should be drawn from this study. First, tools such as Heart Aware could be a cost-effective way to collect valuable CHD risk factor data. Researchers should begin to think about leveraging such technology by partnering with private sector firms to improve public health datasets. Such efforts can only improve public health surveillance, which is positive for researchers, policymakers, private sector firms, and community-dwelling adults. However, additional recruitment methodologies should be employed (in addition to the Internet) to reduce selection bias. Second, this research confirms the continuing need to educate community-dwelling adults about the need to understand their CHD risk factors. This is especially true regarding HDL-C and LDL-C. Finally, this research raises questions about how to use stratification of CHD risk factor agreement by 10-year CHD risk as a clinical strategy. Very few differences in interrater agreement for any CHD risk factor by 10-year CHD risk were identified in this study. Clinicians may want to consider additional strategies to improve CHD risk factor knowledge among those who currently exhibit the greatest chance of a CHD event in the next 10 years.

## Abbreviations

AHA: American Heart Association
BMI: body mass index
CHD: coronary heart disease
CVD: cardiovascular disease
DBP: diastolic blood pressure
DM: diabetes mellitus
HDL-C: high-density lipoprotein cholesterol
LDL-C: low-density lipoprotein cholesterol
MI: multiple imputation
NHLBI: National Heart Lung and Blood Institute
SBP: systolic blood pressure
TC: total cholesterol
